# Supplementary material for: Mutations in PCYT2 disrupt etherlipid biosynthesis and cause a complex hereditary spastic paraplegia
Source: Brain. 2019 Oct 22;142(11):3382–97. doi: 10.1093/brain/awz291 (PMC6821184; doi:10.1093/brain/awz291)
Supplement: awz291_Supplementary_Data [file awz291_supplementary_data.zip › awz291-Suppl_data/Supplementary_Data4.zip!/Supplementary information 3 - fibroblast lipidomics.zip!/Fibroblast lipidomics/Controle vs Patient2/Statistical plots and data/Random forest.pdf]

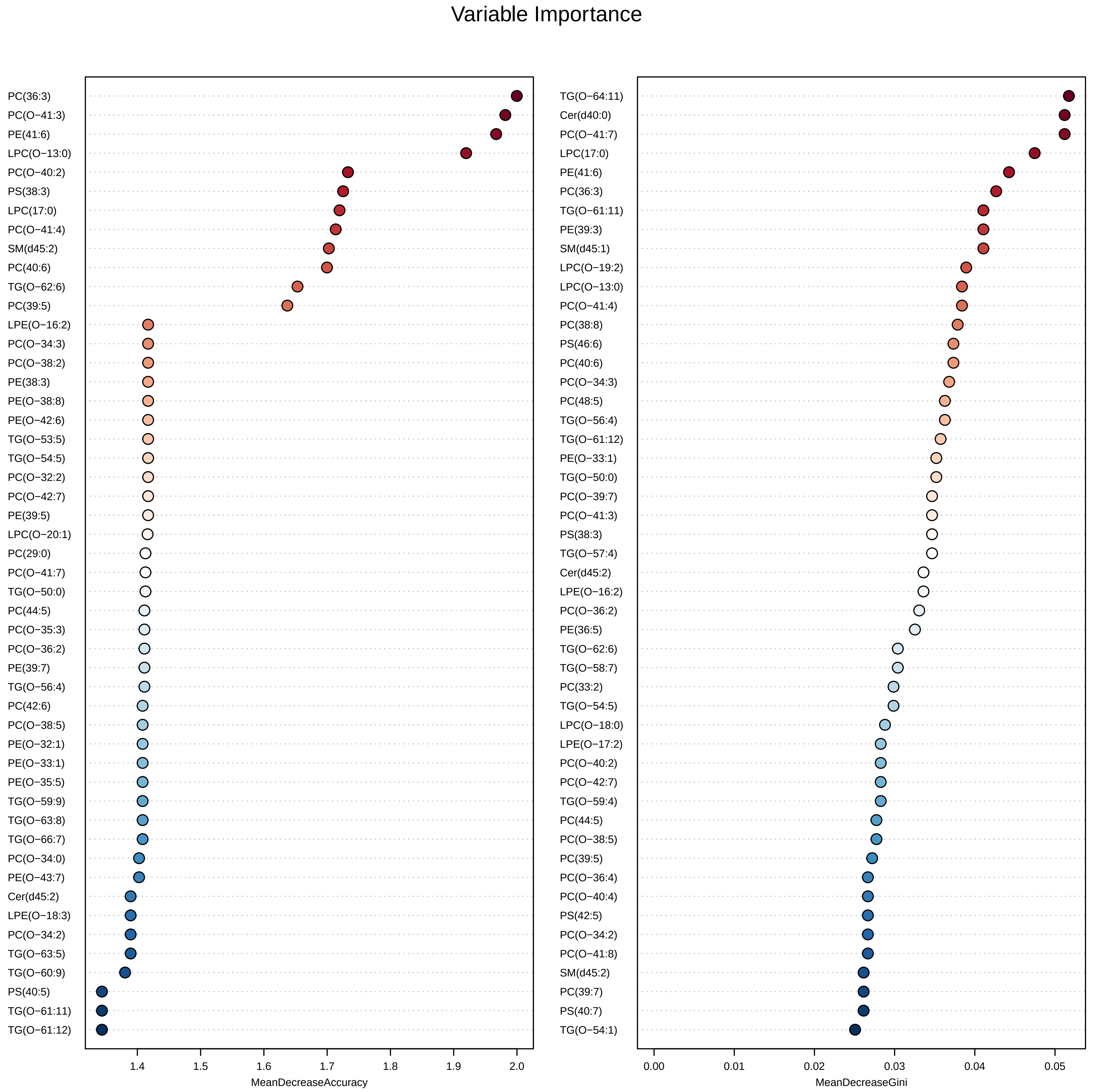
Variable Importance
PC(36:3)
PC(O−41:3)
PE(41:6)
LPC(O−13:0)
PC(O−40:2)
PS(38:3)
LPC(17:0)
PC(O−41:4)
SM(d45:2)
PC(40:6)
TG(O−62:6)
PC(39:5)
LPE(O−16:2)
PC(O−34:3)
PC(O−38:2)
PE(38:3)
PE(O−38:8)
PE(O−42:6)
TG(O−53:5)
TG(O−54:5)
PC(O−32:2)
PC(O−42:7)
PE(39:5)
LPC(O−20:1)
PC(29:0)
PC(O−41:7)
TG(O−50:0)
PC(44:5)
PC(O−35:3)
PC(O−36:2)
PE(39:7)
TG(O−56:4)
PC(42:6)
PC(O−38:5)
PE(O−32:1)
PE(O−33:1)
PE(O−35:5)
TG(O−59:9)
TG(O−63:8)
TG(O−66:7)
PC(O−34:0)
PE(O−43:7)
Cer(d45:2)
LPE(O−18:3)
PC(O−34:2)
TG(O−63:5)
TG(O−60:9)
PS(40:5)
TG(O−61:11)
TG(O−61:12)
1.4 1.5 1.6 1.7 1.8 1.9 2.0
MeanDecreaseAccuracy
TG(O−64:11)
Cer(d40:0)
PC(O−41:7)
LPC(17:0)
PE(41:6)
PC(36:3)
TG(O−61:11)
PE(39:3)
SM(d45:1)
LPC(O−19:2)
LPC(O−13:0)
PC(O−41:4)
PC(38:8)
PS(46:6)
PC(40:6)
PC(O−34:3)
PC(48:5)
TG(O−56:4)
TG(O−61:12)
PE(O−33:1)
TG(O−50:0)
PC(O−39:7)
PC(O−41:3)
PS(38:3)
TG(O−57:4)
Cer(d45:2)
LPE(O−16:2)
PC(O−36:2)
PE(36:5)
TG(O−62:6)
TG(O−58:7)
PC(33:2)
TG(O−54:5)
LPC(O−18:0)
LPE(O−17:2)
PC(O−40:2)
PC(O−42:7)
TG(O−59:4)
PC(44:5)
PC(O−38:5)
PC(39:5)
PC(O−36:4)
PC(O−40:4)
PS(42:5)
PC(O−34:2)
PC(O−41:8)
SM(d45:2)
PC(39:7)
PS(40:7)
TG(O−54:1)
0.00 0.01 0.02 0.03 0.04 0.05
MeanDecreaseGini
